# Estimation of heritability from limited family data using genome-wide identity-by-descent sharing

**DOI:** 10.1186/1297-9686-44-16

**Published:** 2012-05-08

**Authors:** Jørgen Ødegård, Theo HE Meuwissen

**Affiliations:** 1 Nofima, P.O. Box 210, NO-1431 Ås, Norway; 2 Department of Animal and Aquacultural Sciences, Norwegian University of Life Sciences, P.O. Box 5003, NO-1432 Ås, Norway

## Abstract

**Background:**

In classical pedigree-based analysis, additive genetic variance is estimated from between-family variation, which requires the existence of larger phenotyped and pedigreed populations involving numerous families (parents). However, estimation is often complicated by confounding of genetic and environmental family effects, with the latter typically occurring among full-sibs. For this reason, genetic variance is often inferred based on covariance among more distant relatives, which reduces the power of the analysis. This simulation study shows that genome-wide identity-by-descent sharing among close relatives can be used to quantify additive genetic variance solely from within-family variation using data on extremely small family samples.

**Methods:**

Identity-by-descent relationships among full-sibs were simulated assuming a genome size similar to that of humans (effective number of loci ~80). Genetic variance was estimated from phenotypic data assuming that genomic identity-by-descent relationships could be accurately re-created using information from genome-wide markers. The results were compared with standard pedigree-based genetic analysis.

**Results:**

For a polygenic trait and a given number of phenotypes, the most accurate estimates of genetic variance were based on data from a single large full-sib family only. Compared with classical pedigree-based analysis, the proposed method is more robust to selection among parents and for confounding of environmental and genetic effects. Furthermore, in some cases, satisfactory results can be achieved even with less ideal data structures, i.e., for selectively genotyped data and for traits for which the genetic variance is largely under the control of a few major genes.

**Conclusions:**

Estimation of genetic variance using genomic identity-by-descent relationships is especially useful for studies aiming at estimating additive genetic variance of highly fecund species, using data from small populations with limited pedigree information and/or few available parents, i.e., parents originating from non-pedigreed or even wild populations.

## Background

Estimates of additive genetic variance are commonly based on data from large pedigreed populations incorporating all known relationship information. Additive genetic relationships can be defined as twice the identity-by-descent (IBD) probability of two randomly drawn alleles, which can be estimated from pedigree data. The advantages of these pedigree-based analyses are that they do not require any knowledge about the genetic architecture of the traits and that the additive relationships are easily inferred from a known pedigree. However, these methods also have some major limitations. First, such analyses ignore relationships beyond those included in the known pedigree. Second, the assumed relationships are expected relationships (based on expected sharing of IBD alleles) rather than actual relationships. In fact, the pedigree relationship is exact only under an infinitesimal model [1], i.e., assuming that the additive genetic effects of the quantitative traits are controlled by an infinite number of unlinked loci. Under a more realistic finite-locus model (and assuming that some of the loci are linked), the actual relationships will be distributed around the expectation, with variable relationships among full-sibs and other relatives [2]. By assuming (incorrectly) homogeneous relationships among the same type of relatives (e.g., sibs), in pedigree-based analyses, the genetic (co)variance components are estimated based on between-family variation only, since the Mendelian sampling deviations of non-parents cannot be separated from the residual (or permanent environmental) effects on the same animals [3]. Estimation of genetic variance based on pedigree relationships is further complicated by the fact that common environmental effects may be important for some relatives, especially full-sibs (e.g., maternal environment, rearing environment, litter effects, etc.), which means that the genetic variance must be estimated from covariances among phenotypes of more distant relatives (e.g., half sibs, cousins, etc.).

Due to linkage between loci within the same chromosome, parents tend to pass on long segments of DNA to their offspring. Hence, the "effective" number of segregating loci within a full-sib family will be much lower than the corresponding number for the whole population, even for species with a larger genome. For example, recent reports have indicated that the effective number of segregating loci among full-sib pairs in humans is only about 80 [4,5]. When the effective number of segregating loci is low, the actual relationships among full-sibs vary substantially among sib-pairs. Visscher et al. [5] have estimated that actual relationships among human full-sibs vary from 0.37 to 0.62, and used these relationships to quantify the additive genetic variation of human height based on within-family segregation only, i.e., free from non-genetic factors. In this study, the heritability values were based on more than 3000 sib pairs. With such a large dataset, including numerous families, the main challenge is not to estimate between-family variation, but rather to separate genetic effects from other effects that act on a family level. Visscher et al. [5] pointed out that one limitation of their method was that it required large datasets with densely genotyped individuals. Indeed, for a sib-pair design (twin study), a large number of full-sib pairs would be needed. However, for livestock, aquaculture species and laboratory animals, population structures are usually very different from those in humans, with much larger progeny groups of either full- or half sibs (or both). Therefore, the aim of the current study was to test whether genetic variance could be accurately estimated with relatively small datasets and a limited number of families, using a population structure typical of a high fecundity species (e.g., insects, crustaceans, fish or poultry), and whether the results could also be generalized to species in which only one of the sexes (usually males) has a large reproductive potential (e.g., mammalian livestock).

## Methods

### Simulation study

#### Genomic identity-by-descent relationships

**Figure 1:**
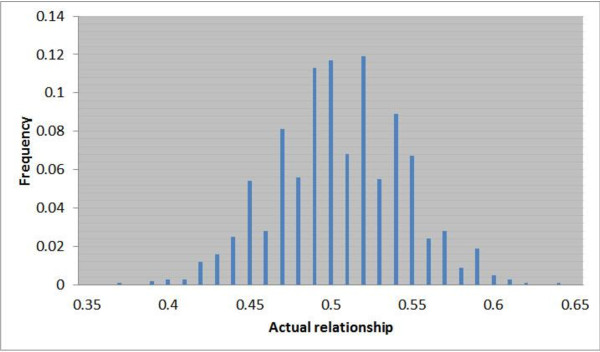
**Example of actual relationships among simulated full-sibs in a single family (*N *= 1000)**.

The IBD relationships were simulated so that they closely resembled the relationships estimated with real data for humans, and thus they were typical of species with relatively large genomes. Variation in IBD sharing was simulated using a model with 80 "effective loci" (*n_e_*) within a family (equivalent to human genome size). Effective loci are defined as the number of independently segregating "loci" that would yield the same standard deviation of the proportion of genome shared among full-sibs as observed in real genomic data from human sib pairs [4]. Hence, an "effective locus allele" is not a specific mutation, but is equivalent to a long haplotype block passed on from parent to offspring. For simplicity, it was assumed that different families were unrelated and that inbreeding was zero. For an "effective locus" *i*, the IBD relationship of two full-sibs was therefore defined as 0 if none of the paternal and maternal "alleles" (haplotype blocks) were IBD, 0.5 if either their paternal or maternal "alleles" were IBD and 1 if both their paternal and maternal "alleles" were IBD. The actual relationship between two full-sibs was then defined as the average relationship across all "effective loci" (i.e., representing the whole genome). An example of the distribution of actual relationships in a large simulated full-sib family is shown in Figure 1. Since all relationships among full-sibs are based on the inheritance of a limited number of "effective loci" (*n_e _*= 80), the actual relationship matrix cannot be of full rank for large size families, which introduces numerical problems in data simulation and analysis. Therefore, the relationship matrix was forced to be positive definite by adding a small positive value (10^-3^) to each diagonal element (sufficiently small to have a neglible effect on the genetic (co)variance structure).

#### Data sets and data structures

Nine data structures were generated, using various numbers of full-sib families (1-10) and individuals (200-1000) with data (Table 1). Furthermore, three scenarios were defined (Table 2), all assuming moderate heritability, but differing with respect to the distribution of genetic variance; either equally distributed over genomic regions (Scenarios 1 and 2) or located in a single region ("one effective locus") only (Scenario 3). Furthermore, Scenario 1 included common environmental family effects in addition to additive genetic effects, while Scenarios 2 and 3 assumed that common environmental effects were absent.

**Table 1:** Simulated data structures

Structure	Animals	Full-sib families	Animals per family
1	200	1	200
2	200	5	40
3	200	10	20
4	500	1	500
5	500	5	100
6	500	10	50
7	1000	1	1000
8	1000	5	200
9	1000	10	100

**Table 2:** Input variance components

Scenario	Additive genetic	Common environment	Residual	Heritability*
1	0.50	0.25	1.00	0.33
2	0.50	0.00	1.00	0.33
3	0.50^†^	0.00	1.00	0.33

*heritability was calculated as genetic variance/(genetic variance + residual variance);

^†^all genetic variance was located at one of the "effective loci"

All combinations of structures and scenarios were run in 50 replicates and the results were averaged over replicates. However, for single-family structures, in which all animals are necessarily within the same family environment, there was no practical difference between Scenarios 1 and 2 (the environmental family effect will be included in the overall mean). Hence, 1200 different datasets were generated and analyzed.

Phenotypes were generated using the following model:

(1) $$y\;=\;1\mu \;+\;Z_{a}a\;+\;Z_{f}f\;+\;e$$

where *μ *is the overall mean, $a\;\sim \;\text{N}\left(0,\;G\sigma_{a}^{2}\right),\;f\;\sim \;\text{N}\left(0,\;I\sigma_{f}^{2}\right),\;e\;\sim \;\text{N}\left(0,\;I\sigma_{e}^{2}\right),$**G **is the actual IBD relationship matrix, **I **is an identity matrix of appropriate size, the **Z**-matrices are appropriate incidence matrices and $\sigma_{a}^{2},\;\;\sigma_{f}^{2}$ and $\sigma_{e}^{2}$ are the additive genetic, common environmental and residual variances, respectively. For Scenarios 1 and 2, **G **was set up over all "effective loci", while for Scenario 3, only the first "effective locus" was used to calculate **G**. The breeding values in **a **were then generated as:

(2) a=Lz

where **L **is a lower triangular Cholesky decomposition of **G**, and **z **is a vector of standard normal deviates of length *N *(number of animals in the dataset). This assumes that (1) genetic variance is evenly distributed across the genome, and (2) gene effects are normally distributed, or that the aggregated effect of many genes, i.e., the breeding values, are approximately normally distributed, even when individual gene effects are not (due to the central limit theorem). It is also assumes that the different founder alleles at an "effective locus" have unique allelic effects, because an "effective locus" contains many genes and thus contains a unique combination of alleles at these genes. All datasets were generated using the MATLAB^® ^software http://www.mathworks.com.

#### Statistic alanalysis

The data sets were analyzed with the general linear model

$$y\;=\;X\beta \;+\;Z_{a}a\;+\;e$$

where **Xβ **includes fixed effects of each family, or in absence of common environmental family effects, the overall mean only. If fitted, common environmental family effects were included as fixed effects due to the fact that the number of families included was very small (and the number of observations per family large) and thus the associated variance was difficult to estimate.

##### Model 1: Genomic IBD animal model

In this model, the additive genetic effects were assumed: $a\;\sim \;\text{N}\left(0,\;G\sigma_{a}^{2}\right),$ where **G **was calculated over all genomic regions, i.e. it was assumed that the inheritance of the DNA segments from parents to offspring could be accurately traced using marker information, and genetic variance was evenly distributed over genome segments (irrespective of the simulation scenario). The genomic IBD animal model is equivalent to a genome-wide gametic model, i.e., a model in which the original gametes received from the sire and dam and their associated actual relationships are reconstructed using genomic data [6]. The relationship between two individuals in the animal model is twice the average of the four gametic relationships (coancestry) for the two individuals, and the animal genetic variance is twice the gametic variance.

##### Model 2: Pedigree-based animal model

This is the classical animal model, assuming $a\;\sim \;\text{N}\left(0,\;A\sigma_{a}^{2}\right),$ where **A **is the numerator relationship matrix (inferred from the pedigree). Furthermore, since the classical model uses only between-family variation to estimate additive genetic effect variance, environmental effects common to full-sibs could not be included in the model for this simple data structure (irrespective of whether they were present or not).

For both models, variance components were estimated with restricted maximum likelihood methodology using the ASREML software package [7].

##### Selective genotyping

The genomic IBD model assumes that all animals are genotyped with a sufficiently dense marker map covering the entire genome. However, in some studies, selective genotyping of phenotypically extreme (high/low) animals within each family may be used to save costs. This may be a useful approach for QTL (Quantitative Trait Loci) detection, but our aim was to evaluate whether such data could be used to estimate quantitative genetic variation as well. In these analyses, we assumed a single family with 200, 500 or 1000 full-sibs, and for which only individuals with phenotypes deviating more than one residual standard deviation from the mean were genotyped. However, because including only the genotyped (phenotypically extreme) animals in the analysis would probably yield overestimated variance components, the non-genotyped animals were also included in the analysis. For the analyses, genomic IBD relationships among genotyped individuals were combined with pedigree relationships of non-genotyped individuals in a common relationship matrix [8,9].

## Results

**Figure 2:**
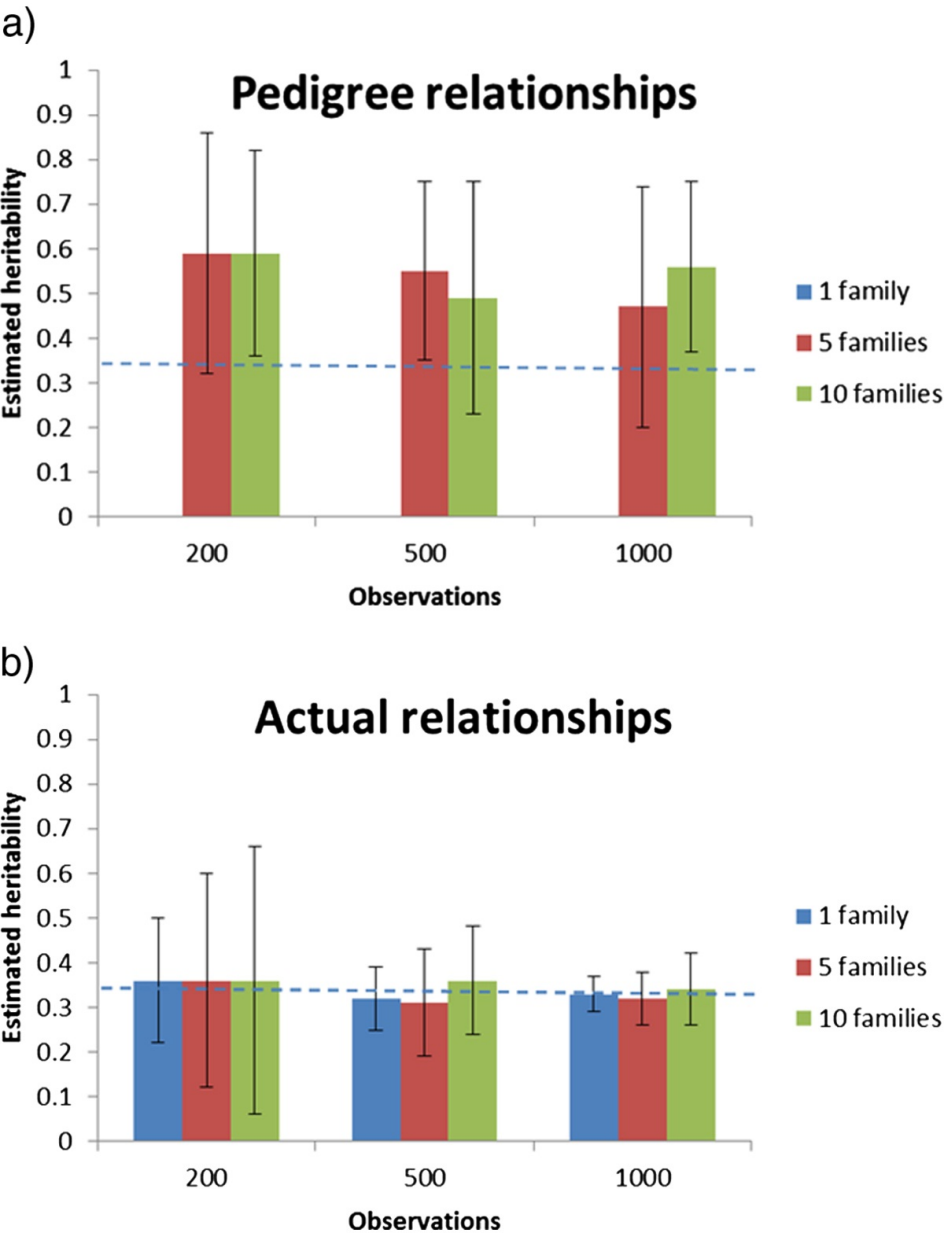
**Across-replicate averages of estimated heritability (with between-replicate standard deviations of the estimates) by total number of observations using a classical animal model (a) and a genomic IBD animal model (b) with 1, 5 or 10 families for Scenario 1**. The dotted line represents the true input heritability.

**Figure 3:**
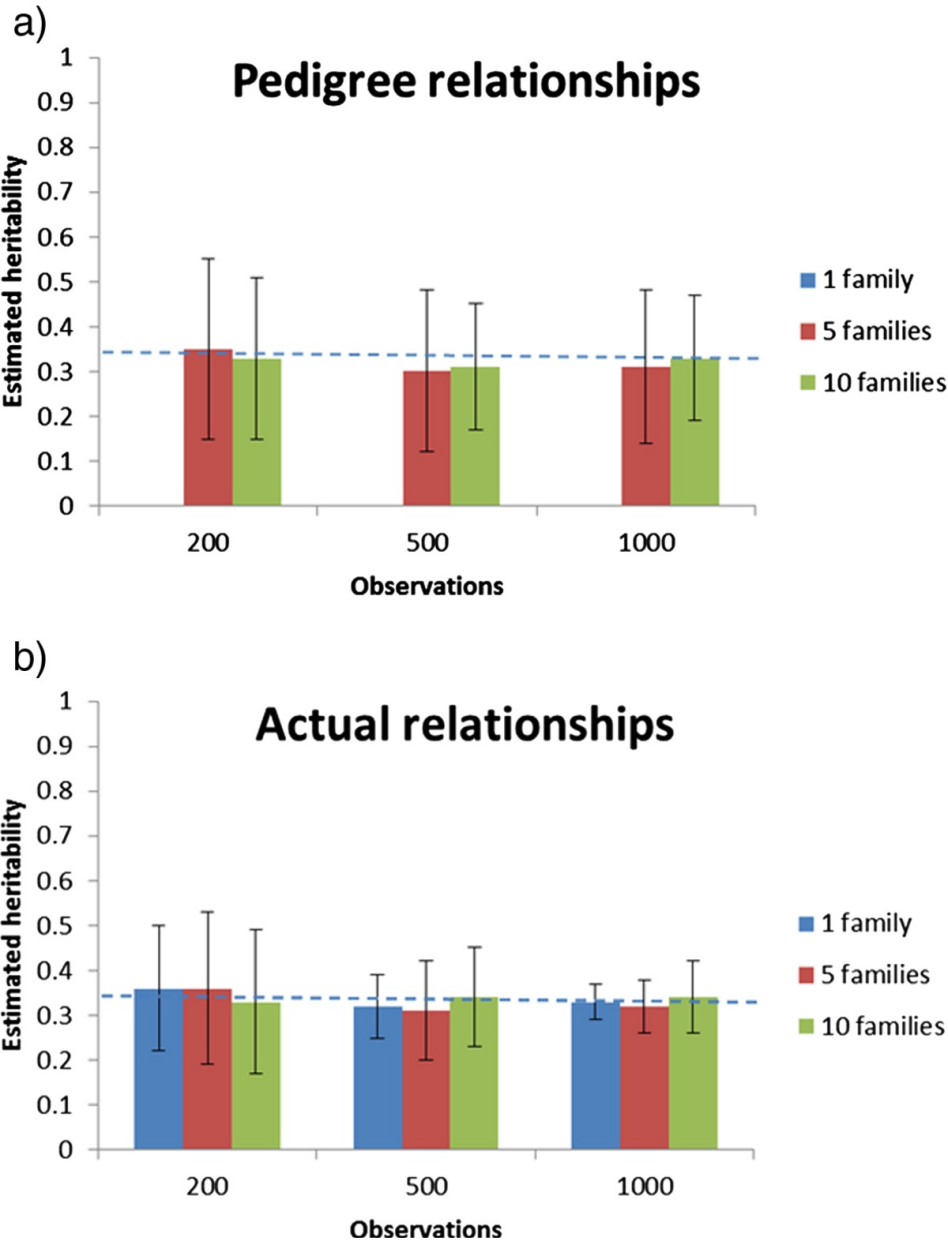
**Across-replicate averages of estimated heritability (with between-replicate standard deviations of the estimates) by total number of observations using a classical animal model (a) and a genomic IBD animal model (b) with 1, 5 or 10 families for Scenario 2**. The dotted line represents the true input heritability.

The estimated heritabilities (across-replicate means and standard deviations) for the different structures under Scenarios 1 and 2 are presented in Figures 2 and 3, respectively. For the classical pedigree-based analyses, the data structure did not make it possible to separate permanent environmental effects common to full-sibs from genetic effects, since both factors are estimated from between-family variation only (and no other relatives than full-sibs were present). Hence, the estimated heritability in the classical model was biased by the common environmental component, resulting generally in over-estimated genetic variance. Furthermore, when the number of families included in the dataset was low, the estimates also varied substantially from replicate to replicate. For the one-family designs, no between-family variation existed, and therefore, by definition, genetic variance could not be estimated with a classical pedigree-based model. However, for all the designs, the genomic IBD model was able to estimate genetic variance, due to the fact that the model inferred genetic variance from within-family variation, and multiple families were therefore not needed. Moreover, even with multiple families, the heritability estimates were unbiased and much more accurate than with the classical model. Furthermore, precision of the heritability estimate in the IBD model increased with increasing family sizes and were most precise for single-family designs (i.e., largest family size for a given number of observations). For the latter design, heritabilities were estimated with moderate to high precision even with the smallest datasets (200 animals).

When assuming no common environmental variance (Scenario 2), the pedigree-based analyses were also unbiased but they were less precise than the genomic IBD analyses (Figure 3). As expected, if common environmental effects were not included in the data, the precision of the estimated heritability was improved, in particular for the smallest datasets using the classical model, while the precision of the IBD model was unaffected for the largest datasets (1000 individuals). The differences between the two models were most pronounced with larger datasets with a few families. For the IBD genomic model, within-family variation dominated estimation of genetic variance, and thus reducing family sizes to give room for more families led to more imprecise estimates of genetic variance.

**Figure 4:**
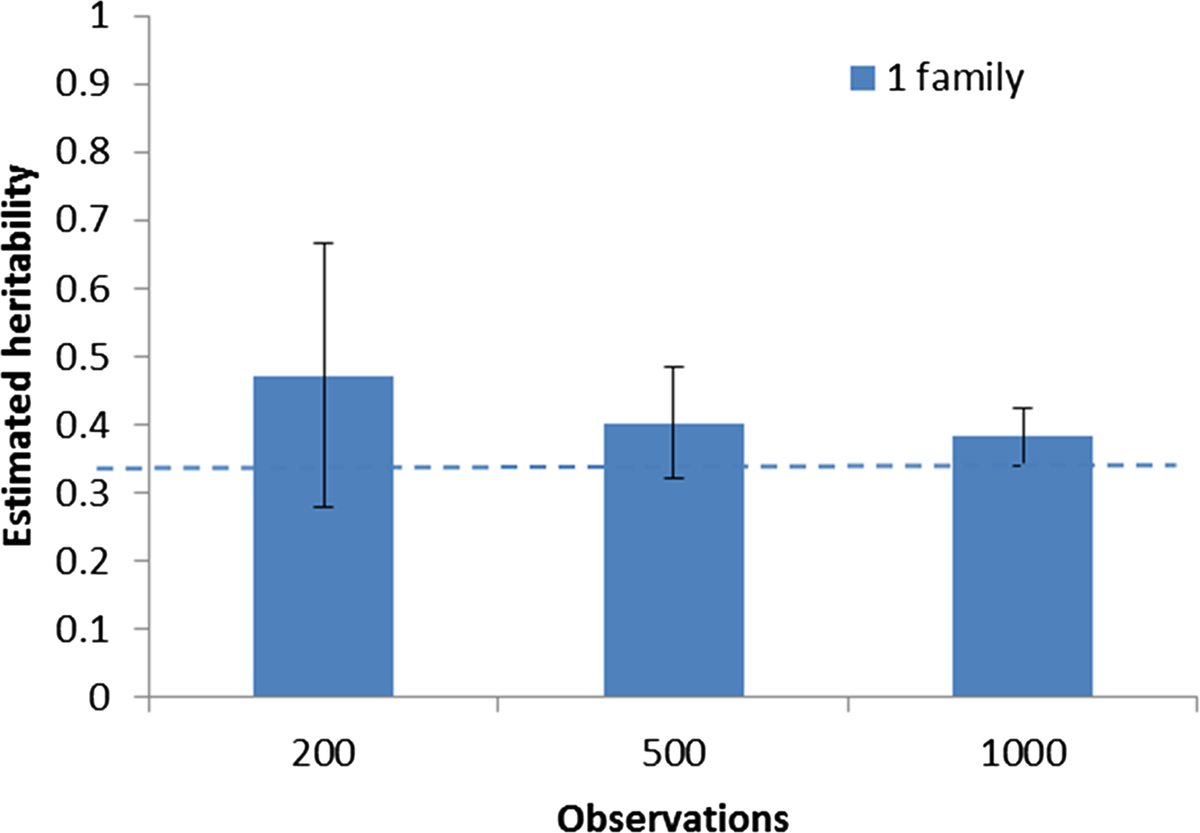
**Across-replicate averages of estimated single-family heritability (with between-replicate standard deviations of the estimates) by total number of observations using a genomic IBD animal model with selective genotyping**. The dotted line represents the true input heritability.

For selectively genotyped data, the genomic IBD model was also able to estimate the genetic variance based on a single large (*N *= 1000) family. However, single-family estimates based on smaller samples (200 or 500) tended to be overestimated, and the precision of the estimates were reduced compared to that with full genotyping (Figure 4).

**Figure 5:**
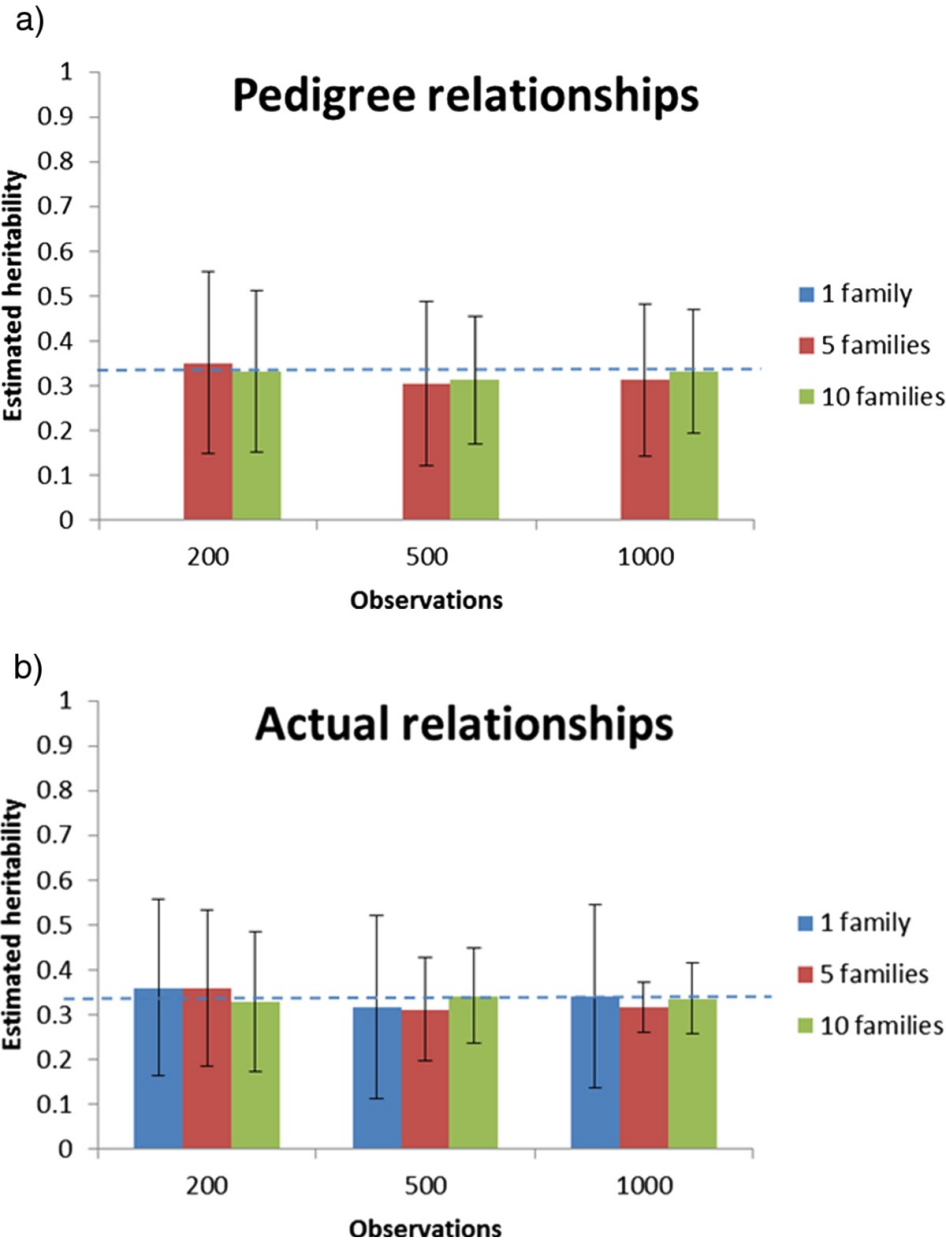
**Across-replicate averages of estimated heritability (with between-replicate standard deviations of the estimates) by total number of observations using a classical animal model (a) and a genomic IBD animal model (b) with 1, 5 or 10 families for Scenario 3**. The dotted line represents the true input heritability.

If all the genetic variance was located in only one "effective locus", and no common environmental variance existed (Scenario 3), estimation of heritability was still unbiased for both the genomic IBD and the pedigree-based (more than one family) methods (Figure 5). With larger datasets (500-1000 individuals), the genomic IBD method was more precise, but the two methods were equally imprecise for the smallest datasets, and, in contrast with the earlier results, the single-family design yielded highly imprecise results with the genomic IBD model.

## Discussion

This study shows that tracing genomic IBD relationships using genomic information has clear advantages, not only for prediction of individual breeding values [10] but also for estimation of genetic (co)variance components. Both the current and earlier studies have shown that genetic variance can be estimated based on within-family variation. In contrast, estimation of genetic variance in a classical genetic analysis is based only on between-family variation. Hence, for the latter, it is imperative that genetic and non-genetic family effects are properly separated by the model, which puts major limitations on the usefulness of family data, e.g., resemblance among full-sibs may also be due to similarities in the environment. Furthermore, for an accurate estimation of genetic variance in a classical model, many families must be included in the study and selection of data should be avoided. However, by using actual IBD sharing among sibs instead of expected relationships, genetic variation can be quantified solely from within-family variation [5], which also facilitates proper separation of genetic and non-genetic family effects (as the latter do not affect within-family variation). The current study shows that with the genomic IBD approach, genetic variance can be accurately inferred from a single family, and for a given number of observations, including more families gives less accurate results (even in the absence of common environmental effects). For a classical analysis (and in absence of common environmental effects), Falconer and Mackay [11] showed that the optimal family size for a specific number of observations under a full-sib design was *n *= 2/*h*^2^. However, using the genomic IBD approach, this formula is no longer valid. For a genomic IBD analysis, including only a single large family will maximize the precision of the predicted Mendelian sampling deviations of all family members, and there is also no need to separate genetic and environmental family effects. Furthermore, the number of families is necessarily lower than the number of individuals, and prediction of Mendelian sampling deviations is therefore more informative than prediction of family means with respect to genetic variance. Estimation of genetic variance from family means (classical model) is also sensitive to selection of data (both within and among families), and failure to account for this is expected to give downward biased estimates of the genetic variance. In populations undergoing artificial selection, parents of the phenotyped animals are usually selected, and unbiased genetic analysis requires that the selection history is properly included in the data, i.e., the analysis should involve data on multiple generations, which is not always available. However, selection among parents will have little impact on the within-family genetic variance (in absence of inbreeding). Since the genomic IBD model uses mainly within-family variation, it is expected to be more robust to selection among parents.

Even with selective genotyping, the genomic IBD model could estimate genetic variance relatively precisely for large families (*N *= 1000), although there was a tendency towards overestimated heritability values and less precise results for smaller family sizes. This may be explained by the fact that only the phenotypically most extreme individuals are genotyped and only these are informative with respect to partitioning of residual and Mendelian sampling variances.

The current study assumes that inheritance of the haplotype blocks from parents to offspring is known. In real data, this is never the case but we may observe genome-wide marker genotypes and this information can be used to trace inheritance of the haplotype blocks. Furthermore, since the number of recombinations per gamete is limited, sharing of haplotype blocks within a family can be estimated with a high degree of accuracy, even with sparsely distributed genome-wide markers [12]. Reconstructing paternal and maternal haplotype blocks is equivalent to reconstructing the original gametes received from the sire and dam, making the genomic IBD animal model and a genomic IBD gametic model equivalent.

In species with a low reproductive potential, the proposed single-family mating design is of little relevance, since large full-sib groups cannot be produced. However, some species have a high reproductive potential among males, while the reproduction of females is often limited (e.g., in mammalian livestock). For such species, a sire gametic model may be more relevant. In such a model, the sires' gametes are reconstructed and the actual relationships between them estimated. Genetic variance can then be estimated from variation among the sires' gametes (half the within-family genetic variance), rather than variation among individuals (sires' and dams' gametes). In this model, genetic variation due to the dams' gametes will be included in the random residual term and genetic variance may be estimated from samples of offspring (e.g., daughters of dairy bulls).

The proposed method can also be generalized to IBD tracing in more complex pedigrees, i.e., beyond a single generation, allowing information from various types of relatives to be exploited by linkage analysis e.g., [13]. More distant relatives are generally less related but their relationships are also expected to deviate more from their expected values [14]. Hence, distant relatives may provide additional value to estimate genetic variance, especially for populations with smaller full- and half-sib groups. However, tracing haplotype blocks over multiple generations will be more challenging (shorter DNA blocks due to more recombination) and will require denser marker maps for accurate tracing compared with the one-generation (sib) approach.

The proposed method will underestimate the total genetic variance in cases where a fraction of the genome is not covered by the markers, i.e., if some of the "effective loci" are not accounted for in the **G **matrix. For instance, if a fraction *q *of the genome is not covered by markers, the total variance will also be underestimated by a fraction *q *when the single full-sib family design is used. When the design contains several families, the between-family variances are quite accurately predicted, even if part of the genome is not covered by markers, which will recover some of the underestimation. The underestimation may be completely recovered by including a polygenic effect in the model, which has a covariance structure equal to the pedigree-based relationship matrix, requiring several families of data.

Scenarios 1 and 2 in the current study assumed that genetic variance is evenly distributed over genomic regions, as assumed in the genomic BLUP (GBLUP) model [10]. The main difference between the GBLUP model and the genomic IBD model is that the first model uses identity-by-state (IBS) relationships, while the latter uses IBD relationships (based on marker alleles traced back to a common ancestor). The assumption that genetic variance is distributed evenly across genomic regions has been shown to be an appropriate approximation for a number of traits [15,16]. However, there are also examples of the opposite assumption, e.g., genetic variation in resistance against infectious pancreas necrosis in Atlantic salmon seems largely controlled by a single major QTL [17,18]. For the latter type of traits, some of the underlying assumptions of both the pedigree-based and genomic IBD models are violated. First, within-family genetic variance will vary greatly among families, depending on the actual parental genotypes ("effective alleles") for the genomic region that primarily affects the trait (although it will still on average be $\frac{1}{2}\sigma_{a}^{2}$); in the example on Atlantic salmon, the within-family genetic variance will depend on whether or not the parents segregate for the major QTL. Second, IBD relationships in the most important linkage group(s) will dominate genetic covariance between relatives, not the overall genomic or expected (pedigree-based) IBD relationships. Still, even for such data, the genomic IBD model could estimate genetic variance more accurately than the classical pedigree-based analysis. Hence, although the genomic IBD relationships are not necessarily representative of the genetic covariance structure among sibs in this situation, they are still more informative than the pedigree-based relationships. In this setting, the differences between the classic pedigree and genomic IBD models increased with the size of the dataset (no practical difference with 200 individuals but a substantial difference with 1000 individuals). However, estimation of genetic variance within a single family was, as expected, highly prone to sampling effects. In Scenario 3, the real number of different breeding values represented within a single full-sib family is actually limited to four (two "effective alleles" per parent), which explains the large between-replicate deviations in the estimated heritability. Thus, in real data, for which the underlying genetics of the trait is generally unknown, it is recommended to use more than one family for quantitative genetic analysis, even when applying the genomic IBD approach.

## Conclusions

The proposed genomic IBD method is particularly relevant for quantitative genetic studies aiming at estimating additive genetic variance of highly fecund species, using data on populations with limited pedigree information and/or few available parents. For example, genetic variance may be estimated based on a few full-sib-families with parents sampled from the wild or from non-pedigreed domesticated populations. In principle, the genomic IBD model (or equivalent gametic model) requires only a single large family for proper and accurate estimation of heritability for quantitative traits. In contrast, classical pedigree-based estimation requires the establishment of a sizeable pedigreed population consisting of numerous full- and (preferably) half-sib families to produce estimates with acceptable accuracy. Furthermore, the proposed genomic IBD model is expected to be less affected by selection among parents and will facilitate the separation of genetic and non-genetic family effects (e.g., effects of common rearing).

## Competing interests

The authors declare that they have no competing interests.

## Authors' contributions

JØ was mainly responsible for conception and design of the study, data analysis and writing of the manuscript. THEM contributed to the design of the study and to writing and revision of the manuscript. Both authors read and approved the final manuscript.
